# Biomimetic astragaloside-IV-loaded mesoporous silica nanoparticles for treatment of dilated cardiomyopathy

**DOI:** 10.3389/fphar.2026.1790522

**Published:** 2026-04-09

**Authors:** Chenghua Wang, Lu Lv, Tao Long, Tiantian Xie, Junqing Qi, Huiwen Pan, Yifen Fang, Yangyong Sun

**Affiliations:** 1 Department of Cardiology, Affiliated People’s Hospital of Jiangsu University, Zhenjiang, China; 2 Department of Cardiothoracic Surgery, Affiliated People’s Hospital of Jiangsu University, Zhenjiang, China; 3 Jiangsu Medical College, Yancheng, China; 4 Department of Cardiology, The Affiliated Traditional Chinese Medicine Hospital, Guangzhou Medical University, Guangzhou, China

**Keywords:** astragaloside IV, cardiac dysfunction, dilated cardiomyopathy, macrophage membrane, mesoporous silica nanoparticles

## Abstract

Dilated cardiomyopathy (DCM) is currently the most prevalent form of cardiomyopathy; it poses a severe threat to human health and is characterized by poor prognosis and high mortality. In this study, we prepared macrophage-membrane-coated and astragaloside-IV-loaded mesoporous silica nanoparticles (AS@MSN/TA/MM) and assessed their therapeutic effects *in vitro* and *in vivo*. The designed AS@MSN/TA/MM were spherical and had an average particle size of approximately 234 nm, a zeta potential of −24.5 mV, a drug loading capacity (LC) of 9.5% ± 0.04%, and an encapsulation efficiency (EE) of 98.9% ± 0.1%. The particles showed remarkable colloidal stability with phosphate-buffered saline or fetal bovine serum *in vitro*; they also improved the doxorubicin hydrochloride (DOX)-induced oxidative stress of H9C2 cells and promoted cell proliferation. In the DOX-induced DCM mouse model, AS@MSN/TA/MM ameliorated cardiac dysfunction and hypertrophy, alleviated cardiomyocyte fibrosis, as well as suppressed serum levels of aldosterone, angiotensin II, and suppression of tumorigenicity 2. The present work sheds light on a promising therapeutic strategy for DCM and provides a robust platform for developing advanced astragaloside-IV-based nanotherapeutics.

## 1 Introduction

Dilated cardiomyopathy (DCM) is a major subtype of cardiomyopathy characterized by progressive left-ventricular dilation and impaired systolic function (Harding et al., 2023; Merlo et al., 2018). In the absence of etiology-specific treatments, DCM is associated with poor prognosis and a 10-year mortality of approximately 40%, which pose a substantial global cardiovascular health burden (Gigli et al., 2025; Heymans et al., 2023). The current therapeutic strategies for DCM are focused on managing the symptoms, reducing the risk of sudden cardiac death, and ameliorating the structural and electrical complications associated with disease progression. However, these measures are suboptimal for halting disease progression and reversing myocardial remodeling, highlighting the urgent need for innovative and targeted therapeutic approaches.

Astragaloside IV (AS) is a primary bioactive saponin extracted from the traditional Chinese herb *Astragalus membranaceus*; it has attracted significant attention in cardiovascular research owing to its well-documented cardioprotective properties, such as oxidative stress attenuation, myocardial inflammation inhibition, and cardiomyocyte survival promotion (Liang et al., 2023; Tang et al., 2018; Lu et al., 2018). However, the clinical applications of AS are severely hindered by its inherent limitations, including poor aqueous solubility, low bioavailability, and non-specific tissue distribution, which collectively compromise the therapeutic efficacy.

To address these challenges, nanocarrier-based drug-delivery systems have emerged as a promising strategy to optimize the pharmacokinetic and pharmacodynamic profiles of bioactive compounds. Mesoporous silica nanoparticles (MSNs) are among the most prominent nanoplatforms available today owing to their high specific surface area, tunable mesoporous structure, excellent biocompatibility, and outstanding drug loading capacity (LC) (Xu et al., 2023; Djayanti et al., 2023); these features make them ideal carriers for encapsulating and delivering hydrophobic drugs like AS. However, bare MSNs have often been reported to have issues such as premature drug leakage, insufficient *in vivo* stability, and lack of active targeting ability that restrict their therapeutic potential in the treatment of DCM.

Surface modification of MSNs is a feasible approach for overcoming the aforementioned drawbacks. Tannic acid (TA) is a natural polyphenol with abundant catechol and galloyl groups, which exhibits strong adhesive properties that enable the formation of stable coatings on the surfaces of nanoparticles via non-covalent interactions (e.g., hydrogen bonding and electrostatic attraction) (Wang et al., 2022a). Furthermore, TA possesses inherent antioxidant, anti-inflammatory, and cardioprotective activities (Jing et al., 2022) that could work synergistically with AS to enhance the cardioprotective effects in DCM to some extent.

In recent years, cell-membrane-coated nanoparticles have revolutionized targeted drug delivery by leveraging the unique biomimetic features of the source cells to enhance biocompatibility, improve immune evasion, prolong circulation, and support tissue-specific homing capabilities; hence, they are considered as appealing drug delivery platform and have demonstrated promising results in various disease models (Oroojalian et al., 2021; Shu et al., 2022; Zhang et al., 2018). Macrophages are some of the key regulators of cardiac inflammatory responses that exhibit natural tropism toward inflamed myocardial tissues; this characteristic can be exploited to enhance the cardiac targeting efficacies of nanocarriers (Park et al., 2020). Specifically, macrophage membrane (MM)-encapsulated nanoparticles have been shown to exhibit inflammatory-site-homing properties owing to their characteristic antigenic markers (Wu et al., 2022).

In the present work, we designed and fabricated a novel multifunctional nanoplatform involving MM-coated AS-loaded MSNs (AS@MSN/TA/MM) for the treatment of DCM. The formulation was thoroughly characterized and evaluated through both *in vitro* and *in vivo* studies. The findings of this study are expected to provide a new therapeutic strategy for DCM and lay the foundation for the clinical application of AS-based nanomedicines.

## 2 Materials

### 2.1 Reagents

Cetyl-trimethylammonium bromide (CTAB), tetraethoxysilane (TEOS), and doxorubicin hydrochloride (DOX) were obtained from Aladdin Chemistry Co., Ltd. (Shanghai, China). As was purchased from Macklin Biochemical Technology Co., Ltd. (Shanghai, China). Hematoxylin and eosin (H&E) stain was supplied by Shanghai Jingke Chemical Technology Co., Ltd. (Shanghai, China). Masson’s trichrome and wheat germ agglutinin (WGA) were purchased from Wuhan Servicebio Technology Co., Ltd. (Wuhan, China). CCK-8 and DAPI were provided by Beyotime Biotechnology Co., Ltd. (Shanghai, China). Calcein AM/propidium iodide (PI) was obtained from Yuanye Bio-Technology Co., Ltd. (Shanghai, China).

### 2.2 Cells

H9C2 cells and leukemia-expressing mouse macrophages (RAW264.7 cells) were obtained from the Chinese Academy of Sciences (Shanghai, China). Both cell lines were cultured in Dulbecco’s modified Eagle’s medium (DMEM) supplemented with 10% fetal bovine serum (FBS), 1% streptomycin (50 U·mL^−1^), and penicillin (50 U·mL^−1^) in a 5% CO_2_ atmosphere at 37 °C.

### 2.3 Animals

Male BALB/c mice (7–8 weeks old) were purchased from Changzhou Cavens Lab Animal Co., Ltd. (Changzhou, China) and housed in a sterile environment with free access to food and water. The animal experiments were approved by the Experimental Animal Ethics Committee of Jiangsu Medical College (XMLL-2022-047) and conducted in accordance with the guidelines of the National Act on the Use of Experimental Animals (People’s Republic of China).

## 3 Methods

### 3.1 Preparation of AS@MSN/TA/MM

#### 3.1.1 Preparation of MSNs

MSNs were prepared via the sol-gel method according to a previous report with slight modifications (Wang et al., 2022b). In brief, 1.0 g of CTAB was dissolved in 400 mL of deionized water and mixed with 2.5 mL of NaOH (2 M) for 5 min; the mixture was then stirred at 80 °C for 1 h, following which 10 mL of TEOS was added dropwise and allowed to react for 2 h. After centrifugation at 8,000 rpm for 20 min, the precipitates were collected and washed with anhydrous ethanol thrice. The obtained precipitate was next dispersed in a mixture of hydrochloric acid and anhydrous ethanol (1:4, v/v), stirred at 60 °C for 6 h, centrifuged at 8,000 rpm for 5 min, and dried in an oven to obtain the MSNs.

#### 3.1.2 Preparation of AS@MSN and AS@MSN/TA

AS was dissolved in methanol and added dropwise into the MSN solution (mass ratio of AS to MSN = 1:2, w/w); the mixture was stirred overnight and then centrifuged at 1,000 rpm for 10 min. The resulting precipitate was designated as AS@MSN. Next, we added TA to AS@MSN, stirred the mixture for 30 min, and centrifuged the resulting product at 10,000 rpm for 10 min to obtain AS@MSN/TA.

#### 3.1.3 Extraction of MMs

We isolated MMs from the RAW264.7 cells using a slight modification of a method described previously (Wang et al., 2021). The RAW264.7 cell membranes were obtained using the Membrane Protein Extraction kit. In brief, the collected cells were dispersed in the membrane protein extraction buffer solution and cooled in an ice bath for 15 min. Thereafter, the cell suspension was transferred to a glass homogenizer and homogenized approximately 30 times. The obtained mixture was then centrifuged at 1,500 rpm for 10 min at 4 °C to remove any cell debris. Subsequently, the supernatant was collected and centrifuged at 14,000 rpm for 30 min at 4 °C; the precipitate was ultrasonicated for 15 min and then extruded through a 400-nm polycarbonate porous membrane 10 times using an Avestin mini extruder (Avestin, LF-1, Canada). The harvested MM vesicles were stored in water at 4 °C until further use. A bicinchoninic acid (BCA) protein assay was employed to analyze the total protein content in the obtained MMs (Lin et al., 2025).

#### 3.1.4 Preparation of AS@MSN/TA/MM

In brief, the MM vesicles and AS@MSN/TA were mixed in a mass ratio of 1:2 (w/w) and sonicated for 3 min. The mixture was then repeatedly squeeze extruded through the 400-nm polycarbonate membrane 10 times using an Avestin mini extruder (Avestin, LF-1, Canada) and centrifuged at 6,000 rpm for 15 min to harvest the AS@MSN/TA/MM.

### 3.2 Characterization

#### 3.2.1 Morphology, particle size, and zeta potential

For the characterization, the sample was first placed on a copper grid and dried. The morphology of the sample was then observed visually using transmission electron microscopy (JEM-2100F, JEOL, Japan). The particle size and zeta potential were next determined by dynamic light scattering at 25 °C using a Malvern Zetasizer Nano ZS unit (Nano ZS 90, Malvern, United Kingdom).

#### 3.2.2 Drug loading capacity (LC) and encapsulation efficiency (EE)

The standard curve of AS was constructed according to a previously reported procedure (Caijun, 2012; Xie and Wu, 2025). In brief, 25 mg of AS was dissolved in 25 mL of methanol to obtain the control solution with a concentration of 1 mg/mL. Then, aliquots of the control solution (0.2, 0.3, 0.4, 0.5, and 0.6 mL) were mixed with 0.5 mL of anhydrous ethanol, 0.5 mL of 8% vanillin in anhydrous ethanol, and 5 mL of 72% sulfuric acid. Each mixture was heated in water bath at 60 °C for 20 min and immediately cooled in an ice bath before being diluted to a total volume of 10 mL with anhydrous ethanol; the absorbance was then measured at 599 nm to generate a regression equation. Lastly, AS@MSN/TA/MM was centrifuged and the AS content of the supernatant was processed similarly. The drug LC and EE parameters were calculated as follows:

$$\text{EE}\left(\%\right)=\left[\frac{\text{Total}\;\text{amount}\;\text{of}\;\text{drug}-\text{Unencapsulated}\;\text{drug}\;\text{in}\;\text{medium}}{\text{Total}\;\text{amount}\;\text{of}\;\text{drug}}\right]\times 100\%,$$

$$\text{LC}\left(\%\right)=\left[\frac{\text{Total}\;\text{amount}\;\text{of}\;\text{drug}-\text{Unencapsulated}\;\text{drug}\;\text{in}\;\text{medium}}{\;\text{Amount}\;\text{of}\;\text{AS}@\text{MSN}/\text{TA}/\text{MM}}\right]\times 100\%.$$

#### 3.2.3 Stability

AS@MSN/TA/MM was suspended in phosphate-buffered saline (PBS; pH 7.4, 10 mM) or 10% FBS, and measurements were performed in triplicate at different intervals.

### 3.3 Cell proliferation assay

The H9C2 cells were seeded on a 96-well plate at a density of 1 × 10^4^ cells per well. Then, DOX was added and the cells were incubated for 24 h to induce the cardiomyocyte injury model. Next, free AS, AS@MSN, AS@MSN/TA, and AS@MSN/TA/MM were added sequentially to the cells and incubated for another 24 h. Finally, the cells were incubated with CCK-8 for 2 h before being detected at 450 nm using a microplate reader (Spectra Max, Molecular Devices, United States). The cell viability was calculated as follows:

$$\text{Cell viability }\left(\%\right)=\left[\frac{\left(\text{Absorbance}\;\text{of}\;\text{sample}\;\text{group}-\text{Absorbance}\;\text{of}\;\text{blank}\;\text{group}\right)}{\left(\text{Absorbance}\;\text{of}\;\text{normal}\;\text{group}-\text{Absorbance}\;\text{of}\;\text{blank}\;\text{group}\right)}\right]\times 100\%.$$

### 3.4 Calcein AM/PI staining

The H9C2 cells were seeded at a density of 1 × 10^4^ cells per well on 96-well plates, and 20 μM of DOX was added to the cells and incubated for 24 h. Then, AS, AS@MSN, AS@MSN/TA, and AS@MSN/TA/MM were added to the cells sequentially and incubated for another 24 h. Subsequently, calcein AM and PI were added and incubated for 30 min. The fluorescence was observed using an inverted fluorescence microscope (Olympus IX83, China).

### 3.5 Detection of reactive oxygen species (ROS)

The H9C2 cells were seeded at a density of 1 × 10^4^ cells per well on 96-well plates and treated with 20 μM of DOX for 24 h. Then, AS, AS@MSN, AS@MSN/TA, and AS@MSN/TA/MM was added to the cells sequentially and incubated for another 24 h. Next, 10 μM of DCFH-DA was added and incubated for 30 min. The cells were observed using an inverted microscope (excitation: 488 nm; emission: 525 nm). Lastly, quantitative analysis was conducted using a microplate reader (SpectraMax, Molecular Devices, United States).

### 3.6 Pharmacodynamic study

#### 3.6.1 Construction of the DCM mouse model

The DCM mouse model was established using a slight modification of a previously report method (Hullin et al., 2018). The mice were injected with DOX (4 mg/kg) intraperitoneally once a week for four consecutive weeks to induce DCM.

#### 3.6.2 Therapeutic efficacy *in vivo*

The DCM mice were randomly divided into five groups (n = 8 each) as follows: PBS (control), AS, AS@MSN, AS@MSN/TA, and AS@MSN/TA/MM (AS dosage = 3 mg/kg). In the normal group, healthy mice were treated with PBS. All mice were dosed via tail vein injection every other day for a total of five times each.

#### 3.6.3 Echocardiography

The mice were anesthetized via intraperitoneal injection of 1% pentobarbital sodium. Then, the cardiac function was assessed through M-mode echocardiography performed using a Kolo Siliconwave 30 portable ultrasound scanner (Suzhou, China) equipped with a 30 MHz transducer. We acquired measurements of the left ventricular internal diameter at end systole (LVESD), end diastole (LVEDD), ejection fraction (LVEF), and fractional shortening (LVFS) and averaged the values over three sequential cardiac cycles (Zhang et al., 2025).

#### 3.6.4 Histological analysis

Within 24 h after the final dosing, the mice were euthanized by cervical dislocation; we then measured the tibia length (TL) and harvested the heart of each mouse. The heart weight index (HWI) was calculated as the ratio of heart weight (HW) to bodyweight (BW) (mg/g); the ratio of HW to TL was also calculated. The heart tissues were fixed in 4% paraformaldehyde overnight, embedded in paraffin, sectioned into 4-μm slices, and stained with H&E, Masson’s trichrome, and WGA (Zhang et al., 2025; Zhang T. et al., 2024). The tissue sections were imaged under a microscope (IMT-2, Olympus Corp., Tokyo, Japan), and the relative fibrotic area and cross-sectional area of cardiomyocytes were quantified using ImageJ software (National Institutes of Health, Bethesda, MD, United States) (Chang et al., 2025).

#### 3.6.5 Biochemistry determination

Serum samples were harvested from the mice, and the levels of aldosterone (ALD), suppression of tumorigenicity 2 (ST2), and angiotensin II (Ang II) were detected using the corresponding ELISA kits.

### 3.7 *In vivo* imaging

The DCM mice were randomly divided into two groups and were administered a tail vein injection of 100 μL of either free indocyanine green (ICG) or ICG@MSN/TA/MM (ICG concentration = 60 μg/mL). Then, fluorescence images were obtained at 2 h, 6 h, and 12 h after administration. At 12 h post-administration, the mice were euthanized by cervical dislocation, and the hearts were excised for *ex vivo* fluorescence imaging (Ma et al., 2025).

### 3.8 Biocompatibility

The hemocompatibility was evaluated by the hemolysis assay (Zhao et al., 2022). First, fresh blood extracted from rabbit heart was centrifuged at 3,000 rpm for 15 min and purified with normal saline (NS) to obtain erythrocytes. Then, each sample was mixed with 2% erythrocytes (v/v) and incubated for 3 h; next, the mixtures were centrifuged at 3,000 rpm for 15 min, and the absorbance (A) of the sample supernatant was measured at 540 nm using a UV-vis spectrophotometer. Here, PBS (0.01 M, pH 7.4) and deionized water were regarded as the negative and positive controls, respectively. The hemolysis ratio (HR) was calculated as follows: HR (%) = [(A_sample_ – A_negative_control_)/(A_positive_control_ – A_negative_control_)] × 100%.

The H9C2 cells were seeded on a 96-well plate at a density of 1 × 10^4^ cells per well and incubated overnight at 37 °C in an incubator with a 5% CO_2_ atmosphere. Then, the excess culture medium was removed, and AS, AS@MSN, AS@MSN/TA, and AS@MSN/TA/MM were added (AS concentration = 10 μg/mL) and incubated for 24 h. The cells were then washed three times with NS, and CCK-8 was added and incubated for 90 min. Lastly, the absorbance was measured at 450 nm using a multifunctional microplate reader to calculate the cell viability.

### 3.9 Statistical analysis

The data were expressed as mean ± standard deviation (SD) values. Statistical analysis was performed via the two-tailed Student’s t-test and analysis of variance (ANOVA) using GraphPad Prism 10 software. The differences were considered to be statistically significant at *p < 0.05, **p < 0.01, or ***p < 0.001.

## 4 Results and discussion

### 4.1 Synthesis and characterization

**FIGURE 1:**
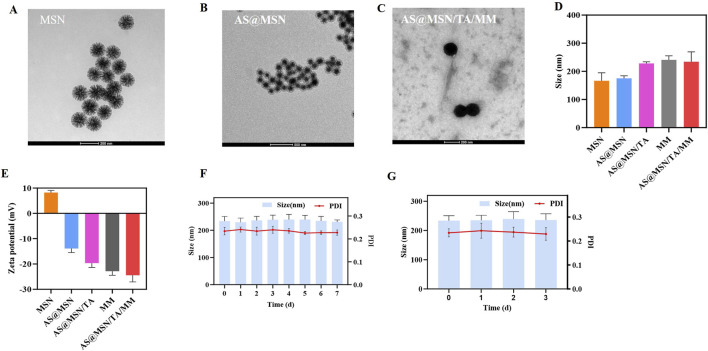
Representative transmission electron microscopy images of **(A)** MSNs (scale bar = 200 nm), **(B)** AS@MSN (scale bar = 500 nm), and **(C)** AS@MSN/TA/MM (scale bar = 200 nm). **(D)** Sizes and **(E)** zeta potentials of the prepared particles. Particle sizes and polydispersity index (PDI) values of AS@MSN/TA/MM after incubation with **(F)** phosphate-buffered saline (PBS) for 7 d at 4 °C (n = 3) and **(G)** 10% fetal bovine serum (FBS) for 3 d at 37 °C (n = 3).

The MSNs exhibited a dendritic morphology with visible mesoporous structures (Figure 1A). After AS loading, adherent substances were observed on the dendritic structures (Figure 1B). Additionally, a bilayer membrane structure was observed on the outermost layer of the AS@MSN/TA/MM, confirming that MM was successfully coated on the nanoparticle surface (Figure 1C).

The average sizes of the MSNs and AS@MSN were 166 nm and 175 nm, respectively (Figure 1D); the size then increased to 234 nm for the AS@MSN/TA/MM upon coating with TA and MM (Figure 1D). The MSNs had a positive zeta potential of 8.3 mV (Figure 1E), which shifted to a negative value for AS@MSN (−13.9 mV) owing to electrostatic adsorption of AS on the surface of the MSN as well as the substances loaded within the mesopores (Figure 1E). Additionally, the zeta potential of AS@MSN/TA/MM (−24.5 mV) was comparable to that of the original MM (−22.9 mV) (Figure 1E). The LC of the particles was 9.5% ± 0.04%, and the EE was 98.9% ± 0.1%. Lastly, AS@MSN/TA/MM exhibited perfect colloidal stabilities in both PBS and 10% FBS, as indicated by the negligible increases in the hydrodynamic diameter and polydispersity index (PDI) (Figures 1F,G).

### 4.2 Cell proliferation

**FIGURE 2:**
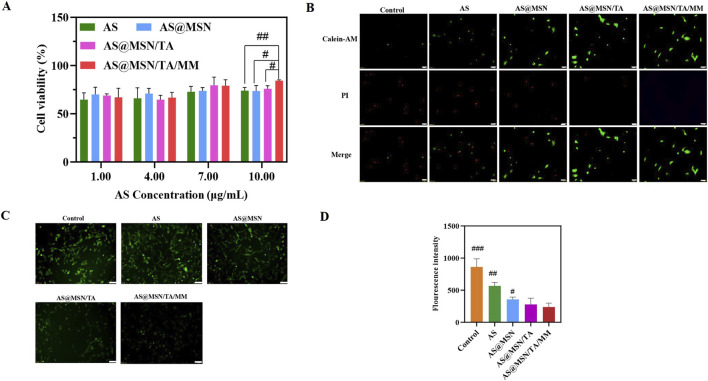
**(A)** Viability of DOX-induced H9C2 cells incubated with different treatment samples at various concentrations for 24 h. **(B)** Labeling of live and dead cells by calcein AM/propidium iodide (PI) for different treatment samples (scale bar = 100 μm). **(C)** Fluorescent images of cells with different treatments and staining with ROS-fluorescent probe DCFH-DA (scale bar = 100 μm). **(D)** Fluorescence intensity quantification by DCFH-DA vs. AS@MSN/TA/MM, ^#^ *p* < 0.05, ^##^ *p* < 0.01, ^###^ *p* < 0.001.

The H9C2 cells showed a dose-dependent cytotoxicity against DOX; accordingly, the H9C2 cells were induced using an optimized dose of 20 µM of DOX (Supplementary Figure S1) (Zhang H. et al., 2024; Che et al., 2022). As shown in Figure 2A, the cell viability was dependent on the dose; the AS@MSN/TA/MM group exhibited superior cell viability than the other groups, indicating that this formulation could significantly alleviate DOX-induced oxidative stress in the H9C2 cells and promote cell proliferation.

### 4.3 Calcein AM/PI staining

In the control groups, very little green fluorescence was observed compared to the obvious red fluorescence, which was attributed to DOX-induced oxidative stress (Figure 2B). Notably, the green fluorescence in the AS@MSN/TA/MM group was significantly higher than those observed in the other groups (Figure 2B). This result confirms that AS@MSN/TA/MM may alleviate DOX-induced oxidative damage to the cardiomyocytes and enhance cardiomyocyte viability.

### 4.4 Cellular ROS detection

As shown in Figures 2C,D, the control group exhibited a greater intensity of green fluorescence, reflecting the high intracellular ROS levels, which are consistent with a previous report (Xiong et al., 2025). DOX-induced cardiotoxicity is associated with oxidative stress (Mantawy et al., 2014). After treatment with different formulations, the green fluorescence intensity decreased (Figure 2C); among all the groups, the AS@MSN/TA/MM group showed the most significant reduction (Figure 2D). Oxidative stress triggers post-translational modifications at mitochondrial complex I glutathionylation (Hao et al., 2026); the ROS-scavenging property of AS@MSN/TA/MM may be linked to the redox homeostasis preservation of complex I.

### 4.5 Pharmacodynamic study

#### 4.5.1 Echocardiography

**FIGURE 3:**
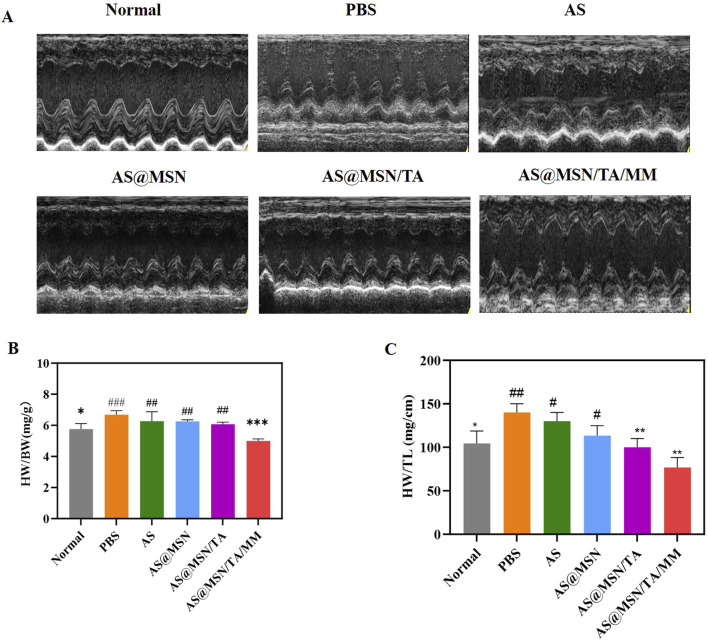
**(A)** Representative echocardiographic images. **(B)** Heart weight index values of the mice after treatment (n = 8). **(C)** Heart weight to tibia length (HW/TL) ratios of the mice after treatment (n = 8). HW, heart weight; BW, bodyweight; TL, tibia length. For PBS, *p < 0.05, **p < 0.01, ***p < 0.001; for AS@MSN/TA/MM, ^##^ *p* < 0.01, ^###^ *p* < 0.001.

As shown in Figure 3A and Table 1, compared to the normal group, the PBS group exhibited significantly increased LVEDD and LVESD values along with significantly decreased LVEF and LVFS levels (*p* < 0.05). Compared to the PBS group, the AS, AS@MSN, AS@MSN/TA, and AS@MSN/TA/MM groups showed varying degrees of reduction in LVEDD and LVESD values as well as increments in the LVEF and LVFS levels. Specifically, the AS@MSN/TA/MM group exhibited better improvement of cardiac dysfunction.

**TABLE 1:** Echocardiographic parameters of the mice in different treatment groups (n = 8).

Group	LVEDD (mm)	LVESD (mm)	LVEF (%)	LVFS (%)
Normal	3.72 ± 0.54*	2.90 ± 0.69*	46.07 ± 13.69*	22.83 ± 8.00*
PBS	3.15 ± 0.18###	1.94 ± 0.08###	69.99 ± 1.19###	38.3 ± 1.12###
AS	2.80 ± 0.02*#	1.53 ± 0.1**#	78.02 ± 4.08**	46.31 ± 3.73**
AS@MSN	2.67 ± 0.57*##	1.45 ± 0.5**	79.43 ± 8.65**	46.97 ± 8.58**
AS@MSN/TA	2.71 ± 0.27*#	1.39 ± 0.10**	81.14 ± 5.15***	48.27 ± 5.44**
AS@MSN/TA/MM	2.22 ± 0.14***	0.94 ± 0.29***	88.75 ± 7.64***	57.99 ± 10.36***

Data are presented as mean ± SD vs. PBS, *p < 0.05, **p < 0.01, ***p < 0.001; vs. AS@MSN/TA/MM, ^#^ *p* < 0.05, ^##^ *p* < 0.01, ^###^ *p* < 0.001.

#### 4.5.2 HWI

As shown in Figure 3B, compared to the normal group, the PBS group displayed higher HWI values (*p* < 0.05); compared to the PBS group, the AS, AS@MSN, AS@MSN/TA, and AS@MSN/TA/MM groups showed varying degrees of reduction in the HWI. Meanwhile, the AS@MSN/TA/MM group exhibited the most significant reduction (*p* < 0.001).

#### 4.5.3 HW/TL ratio

The TL is a stable indicator of linear growth in mice (Cochran et al., 2023). An increase in the HW/TL ratio indicates myocardial hypertrophy, whereas a decrease indicates cardiac atrophy or excessive tibial growth. Compared to the PBS group, the AS@MSN/TA/MM group showed significant decrease in HW/TL (*p* < 0.01) (Figure 3C).

#### 4.5.4 Histological analysis

**FIGURE 4:**
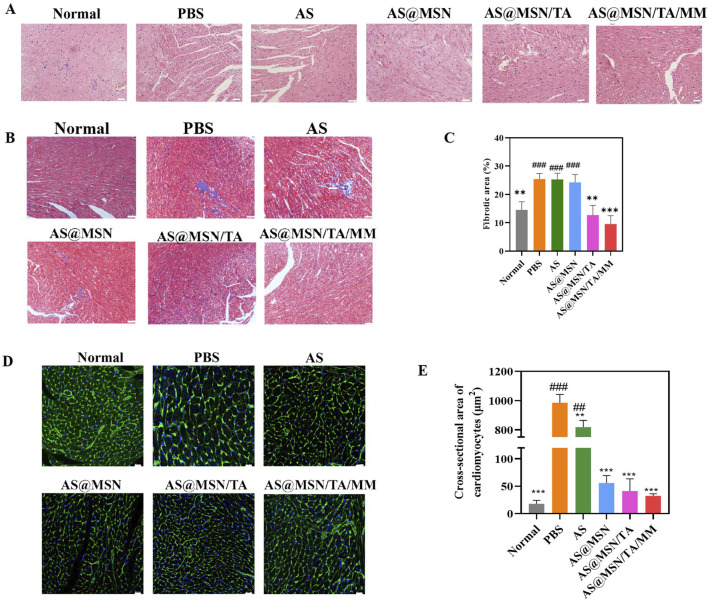
**(A)** Representative images of H&E staining (scale bar = 50 μm). **(B)** Representative images of Masson’s trichrome staining (scale bar = 50 μm). **(C)** Fibrotic area quantification analysis for Masson’s staining (n = 8). **(D)** Representative images of WGA staining (scale bar = 50 μm). **(E)** Cross-sectional area quantification for WGA staining (n = 8). H&E, hematoxylin and eosin; WGA, wheat germ agglutinin. The data are presented as mean ± SD vs. PBS, **p < 0.01, ***p < 0.001; vs. AS@MSN/TA/MM, ^#^ *p* < 0.05, ^##^ *p* < 0.01, ^###^ *p* < 0.001.

The histopathological analyses of the heart tissues using H&E staining are shown in Figure 4A. For the normal group, the cardiomyocytes exhibited regular alignment with preserved nuclear and cytoplasmic architectures (Figure 4A); the PBS group displayed disorganized cardiomyocytes, widened intercellular spaces, loose myocardial fibers, hypertrophy, and necrosis (Figure 4A). Treatment with AS@MSN/TA/MM was observed to significantly attenuate hypertrophic manifestations and necrotic foci (Figure 4A).

The heart sections from different treatment groups stained with Masson’s trichrome are shown in Figure 4B; the sections demonstrate comparable levels of extensive interstitial and perivascular fibrosis with blue collagen deposits in the PBS group, which reduced upon treatment with AS@MSN/TA and AS@MSN/TA/MM. Notably, compared with the PBS group, the fibrotic areas were attenuated significantly in the AS@MSN/TA (*p* < 0.01, Figure 4C) and AS@MSN/TA/MM (*p* < 0.001, Figure 4C) groups. MrgD is a key regulator of collagen I/III production in smooth muscle cells under hypoxic stress and is known to attenuate vascular remodeling and fibrosis via the AKT–MAZ–PIM1 pathway (Zhong et al., 2025). Similarly, AS@MSN/TA/MM may inhibit collagen synthesis in cardiomyocytes.

As shown in Figure 4D, hypertrophied and disorganized cardiomyocytes are observed in the PBS group upon WGA staining, which were improved by AS@MSN/TA/MM. All drug-treated groups exhibited significantly reduced cardiac cross-sectional areas compared to the PBS group (Figure 4E). Interestingly, the AS@MSN/TA/MM formulation demonstrated the most substantial reduction in cross-sectional area, indicating superior therapeutic efficacy in the DOX-induced DCM model (Figure 4E).

#### 4.5.5 Serum ALD, Ang II, and ST2 levels

**FIGURE 5:**
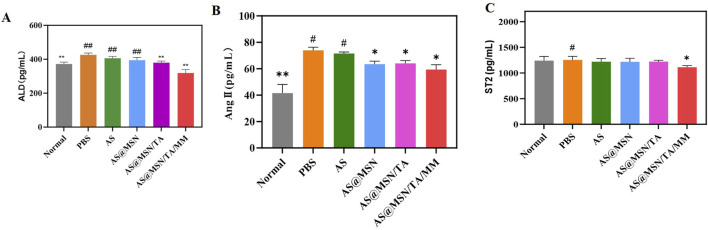
Serum levels of **(A)** aldosterone (ALD), **(B)** angiotensin II (Ang II), and **(C)** suppression of tumorigenicity 2 (ST2) (n = 8). The data are presented as mean ± SD vs. PBS, *p < 0.05, **p < 0.01; vs. AS@MSN/TA/MM, ^#^ *p* < 0.05, ^##^ *p* < 0.01.

As shown in Figure 5, compared to the PBS group, the ALD level decreased in the AS@MSN/TA/MM group (*p* < 0.01); furthermore, both Ang II and ST2 levels were slightly decreased in the AS@MSN/TA/MM group (*p* < 0.05).

The renin–angiotensin–aldosterone system (RAAS) is a major cardiovascular regulator (Zhong et al., 2025; Zhang et al., 2021; Xue et al., 2022), and AS@MSN/TA/MM may interact with the RAAS-related pathways to confer cardioprotection.

AS@MSN/TA/MM likely mediates cardioprotection via PI3K/Akt activation, which orchestrates oxidative stress suppression through Nrf2, autophagy inhibition via mTOR, and profibrotic signaling attenuation through TGF-β/Smad cross talk (Luo et al., 2021; Cheng et al., 2019). In this regard, we intend to provide further validation through phosphoproteomic analysis of the Akt effectors (GSK-3β and FoxO1).

### 4.6 *In vivo* fluorescence imaging

**FIGURE 6:**
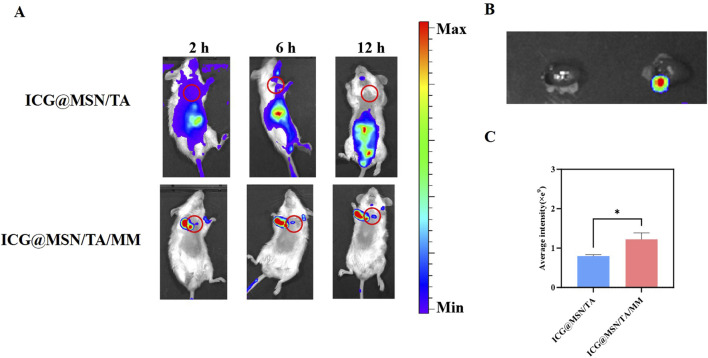
**(A)** Live imaging of dilated cardiomyopathy (DCM) mice and *in vivo* distribution after treatments with ICG@MSN/TA and ICG@MSN/TA/MM (n = 3 each). **(B)** Indocyanine green (ICG) accumulated in the heart 12 h after treatment with ICG@MSN/TA (left) and ICG@MSN/TA/MM (right) (n = 3). **(C)** Quantification of ICG fluorescence intensity from **(B)**, *p < 0.05.

As shown in Figure 6A, weak fluorescence was observed in the heart tissues 2 h post-administration, and the fluorescence intensity increased gradually after 6 h. At 12 h post-administration, the hearts were excised for *ex vivo* fluorescence intensity measurements (Figure 6B). The results show that the ICG@MSN/TA/MM group exhibited strong fluorescence intensity at 12 h, which was significantly higher than that of the ICG@MSN/TA group (Figures 6B,C). This may be attributed to the MM coating, which possesses natural tropism toward inflamed myocardial tissues (Park et al., 2020; Wu et al., 2022).

### 4.7 Biocompatibility

**FIGURE 7:**
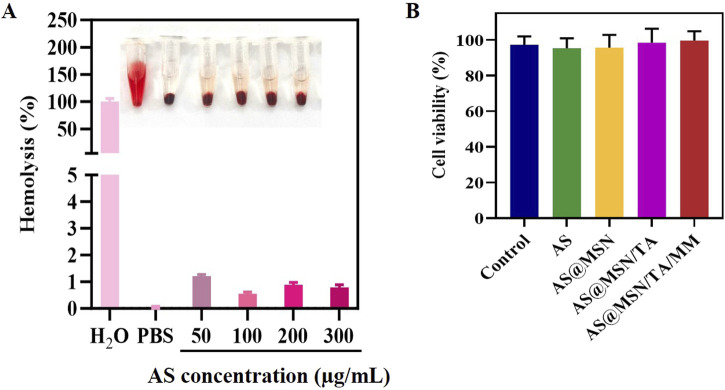
**(A)** Hemolysis ratios of the different treatment samples (n = 3); (inset: hemolytic images). **(B)** Viability of the H9C2 cells after incubation with different treatment samples for 24 h.

Figure 7A showed that the hemolysis rate of AS@MSN/TA/MM was consistently below 5% across all tested concentrations, demonstrating satisfactory biocompatibility. Moreover, the cell viabilities of all groups exceeded 90%, indicating excellent cytocompatibility (Figure 7B).

## 5 Conclusion

Biomimetic AS-loaded MSNs (AS@MSN/TA/MM) were successfully fabricated and demonstrated remarkable therapeutic efficacies against DCM *in vitro* and *in vivo* along with high biocompatibility. These findings support AS@MSN/TA/MM as a promising therapeutic agent and establish a novel strategy for DCM treatment while advancing the development of AS-based formulations. Our future efforts include exploring multifunctional nanosystems, such as those integrating signaling molecules or extracellular matrix components, to enhance the targeting precision and therapeutic efficacy (Yao et al., 2026). We also aim to explore the *in vivo* pharmacokinetic profiles, underlying mechanisms, and clinical translation strategies of these multifunctional nanosystems in future studies.

## Data Availability

The raw data supporting the conclusions of this article will be made available by the authors without undue reservation.
