# Supplementary material for: Biomimetic astragaloside-IV-loaded mesoporous silica nanoparticles for treatment of dilated cardiomyopathy
Source: Front Pharmacol. 2026 Apr 9;17:1790522. doi: 10.3389/fphar.2026.1790522 (PMC13102649; doi:10.3389/fphar.2026.1790522)
Supplement: Supplementary file 1 [file Supplementaryfile1.docx]

**Supporting Information**

1. Cell viability of H9C2 cells incubated with DOX


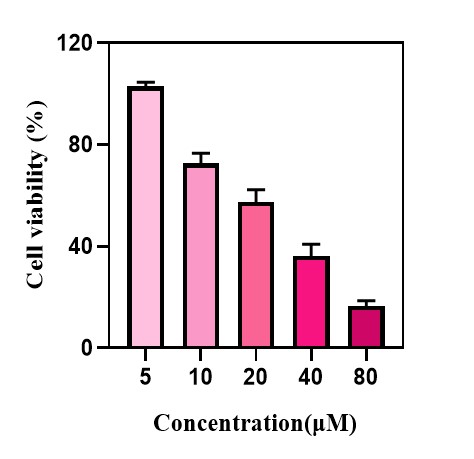


Figure S1. Cell viability of H9C2 cells incubated with DOX at various concentrations for 24 h.
